# Landmark‐based auto‐contouring of clinical target volumes for radiotherapy of nasopharyngeal cancer

**DOI:** 10.1002/acm2.14474

**Published:** 2024-07-29

**Authors:** Carlos Sjogreen, Tucker J. Netherton, Anna Lee, Moaaz Soliman, Skylar S. Gay, Callistus Nguyen, Raymond Mumme, Ivan Vazquez, Dong Joo Rhee, Carlos E. Cardenas, Mary K. Martel, Beth M. Beadle, Laurence Edward Court

**Affiliations:** ^1^ Department of Radiation Physics The University of Texas MD Anderson Cancer Center Houston Texas USA; ^2^ Department of Radiation Oncology The University of Texas MD Anderson Cancer Center Houston Texas USA; ^3^ Department of Radiation Oncology Karmanos Cancer Institute Detroit Michigan USA; ^4^ Department of Radiation Oncology University of Alabama Birmingham Birmingham Alabama USA; ^5^ Department of Radiation Oncology Stanford University Stanford California USA

**Keywords:** auto‐contouring, clinical target volume, nasopharynx cancer

## Abstract

**Background:**

The delineation of clinical target volumes (CTVs) for radiotherapy for nasopharyngeal cancer is complex and varies based on the location and extent of disease.

**Purpose:**

The current study aimed to develop an auto‐contouring solution following one protocol guidelines (NRG‐HN001) that can be adjusted to meet other guidelines, such as RTOG‐0225 and the 2018 International guidelines.

**Methods:**

The study used 2‐channel 3‐dimensional U‐Net and nnU‐Net framework to auto‐contour 27 normal structures in the head and neck (H&N) region that are used to define CTVs in the protocol. To define the CTV‐Expansion (CTV1 and CTV2) and CTV‐Overall (the outer envelope of all the CTV contours), we used adjustable morphological geometric landmarks and mimicked physician interpretation of the protocol rules by partially or fully including select anatomic structures. The results were evaluated quantitatively using the dice similarity coefficient (DSC) and mean surface distance (MSD) and qualitatively by independent reviews by two H&N radiation oncologists.

**Results:**

The auto‐contouring tool showed high accuracy for nasopharyngeal CTVs. Comparison between auto‐contours and clinical contours for 19 patients with cancers of various stages showed a DSC of 0.94 ± 0.02 and MSD of 0.4 ± 0.4 mm for CTV‐Expansion and a DSC of 0.83 ± 0.02 and MSD of 2.4 ± 0.5 mm for CTV‐Overall. Upon independent review, two H&N physicians found the auto‐contours to be usable without edits in 85% and 75% of cases. In 15% of cases, minor edits were required by both physicians. Thus, one physician rated 100% of the auto‐contours as usable (use as is, or after minor edits), while the other physician rated 90% as usable. The second physician required major edits in 10% of cases.

**Conclusions:**

The study demonstrates the ability of an auto‐contouring tool to reliably delineate nasopharyngeal CTVs based on protocol guidelines. The tool was found to be clinically acceptable by two H&N radiation oncology physicians in at least 90% of the cases.

## 1 INTRODUCTION

The incidence of nasopharyngeal cancer (NPC) varies substantially across the globe, with higher rates in endemic areas such as Southeast Asia, and North Africa, and lower rates in North America and Europe.^1^ Curative treatment of non‐metastatic NPC currently necessitates radiation therapy, which requires careful planning and precise treatment delivery to ensure requisite dose to the tumor and to minimize irradiation of adjacent normal tissues.^2^ Over the years, there have been numerous publication of guidelines, protocols, and tools to improve diagnosis, treatment, and prognostication for NPC^3^, ^4^, ^5^, ^6^, ^7^, ^8^, ^9^ that have underscored the importance of meticulous planning for treatment success. Recent technological advancements, especially those involving automation, have improved the consistency and quality of care for patients with diseases such as NPC.^10^, ^11^, ^12^

In countries with a high incidence of NPC, such as China, the abundance of available data facilitates data‐intensive methods of treatment research, such as deep learning to delineate clinical target volumes (CTV). These data provide a foundation for practical training in artificial intelligence techniques for NPC treatment methodologies. Men et al.’s use of a convolutional neural network for CTV delineation was supported by data from 230 patients.^13^ Their work on convolutional neural networks for overall CTV segmentation further benefited from a dataset of 600 patients, facilitating advancements in 3‐dimensional (3D) segmentation and slice labeling.^14^ Xue et al. also showed that a sequential and iterative U‐net (SI‐Net) model outperforms the U‐net model for segmenting CTVs with high‐risk tumors, a feat made possible by including data from 150 patients. As demonstrated by these works, high‐quality datasets with more than 100 patients were instrumental for developing accurate CTV contouring tools for treatment of NPC.^15^ However, such datasets are not always available, especially in regions where NPC is rare, or when a change is made to the contouring guideline.

Our study adopts a landmark‐based approach for auto‐contouring, which requires less training data, contrasting with the current state‐of‐the‐art methods that rely heavily on extensive datasets. In our approach, we used deep learning to initially outline anatomical structures and organs at risk (OARs), but we then applied an algorithm that uses the NRG‐HN001 protocol to identify specific landmarks that guide the definition of CTVs.^16^ This approach is more akin to the contouring process that radiation oncology physicians undergo, and was thus hypothesized to provide reliably accurate targets with more limited datasets

## 2 METHODS

### 2.1 Datasets

Two different cohorts of patients were studied. The first was termed “Cohort 1” and was composed of 99 patients diagnosed with stage I through stage IV NPC at the University of Texas MD Anderson Cancer Center between 2010 and 2020. Non‐contrast CT scans were used for patients in this cohort. Pixel sizes ranged from 0.98 to 1 mm and slice thicknesses ranged from 2 to 2.5 mm.

The second patient cohort was termed “Cohort 2” and was composed of 19 patients treated for NPC at Stanford University per the NRG‐HN001 protocol guidelines. This cohort was used for final testing and represented various NPC disease scenarios, specifically, patients with stages II–IV NPC as well as patients with N1–N3 lymph node status. All CT scans were contrast enhanced. Pixel sizes ranged from 0.98 to 1.36 mm and slice thicknesses between 1.25 and 2.5 mm.

### 2.2 Deep‐learning model

For this research, we selected the 3D U‐net full resolution configuration of the nnU‐net^17^ because of its superior performance in medical segmentation challenges^18^, ^19^ and other studies published in the literature.^20^, ^21^ This model was used to segment 10 distinct structures within the head and neck (H&N) region. These structures were brain, clivus, cricoid, hyoid, left mastoid, right mastoid, maxillary sinus, nasopharynx, left orbit, and right orbit. Cohort 1 was randomly divided into 79 and 20 patients, aligning closely with an 80/20 split, for training and validation purposes, respectively.

During pre‐processing, images were resized, windowed, and z‐normalized using parameters automatically determined by the nnU‐Net framework. All scans were interpolated to a slice thickness of 2.5 mm. Training began with the He initialization and stochastic gradient descent using Nesterov momentum.^22^, ^23^, ^24^ The study employed nnU‐Net's default hyperparameters^17^: initial learning rate 0.01, combined dice and cross‐entropy loss, Stochatic Gradient Descent (SGD) optimizer with Nesterov momentum (μ = 0.99), batch size 2, and 1000 epochs. Data augmentation included rotations, scaling, noise, blur, brightness/contrast adjustments, and mirroring. The framework automatically optimized patch size, network depth, and features per layer. A five‐fold cross‐validation was performed, with ensemble aggregation by softmax probability averaging. Image preprocessing included resampling to median spacing and intensity normalization for CT images. Detailed results for each fold are provided in the supporting information (Figure S1).

### 2.3 Pretrained models

In addition to our primary 3D model used to segment 10 key anatomical structures, our study also included pre‐trained deep learning models. These models, developed in‐house, were designed for segmentation of normal structures in the H&N region. From Rhee et al., this model was used to segment the brainstem, cochlea, mandible, optic nerve, parotid gland, spinal cord, esophagus, and optic chiasm.^25^ From Netherton et al., this model was used to segment the cervical and spinal vertebrae.^26^ From Cardenas et al. and Netherton et al., these models were used to segment the lymph node levels Ia‐V, Ib‐V, II‐IV, and retropharyngeal (RP) lymph nodes.^27^, ^28^ The combination of these pre‐trained models with our primary 3D U‐net model provided a framework for the creation of our CTV automation tool. For more detailed information about these pre‐trained models, please refer to the cited works.^25^, ^26^, ^27^, ^28^

### 2.4 Creation of the automatic CTVs using the protocol guidelines

**FIGURE 1:**
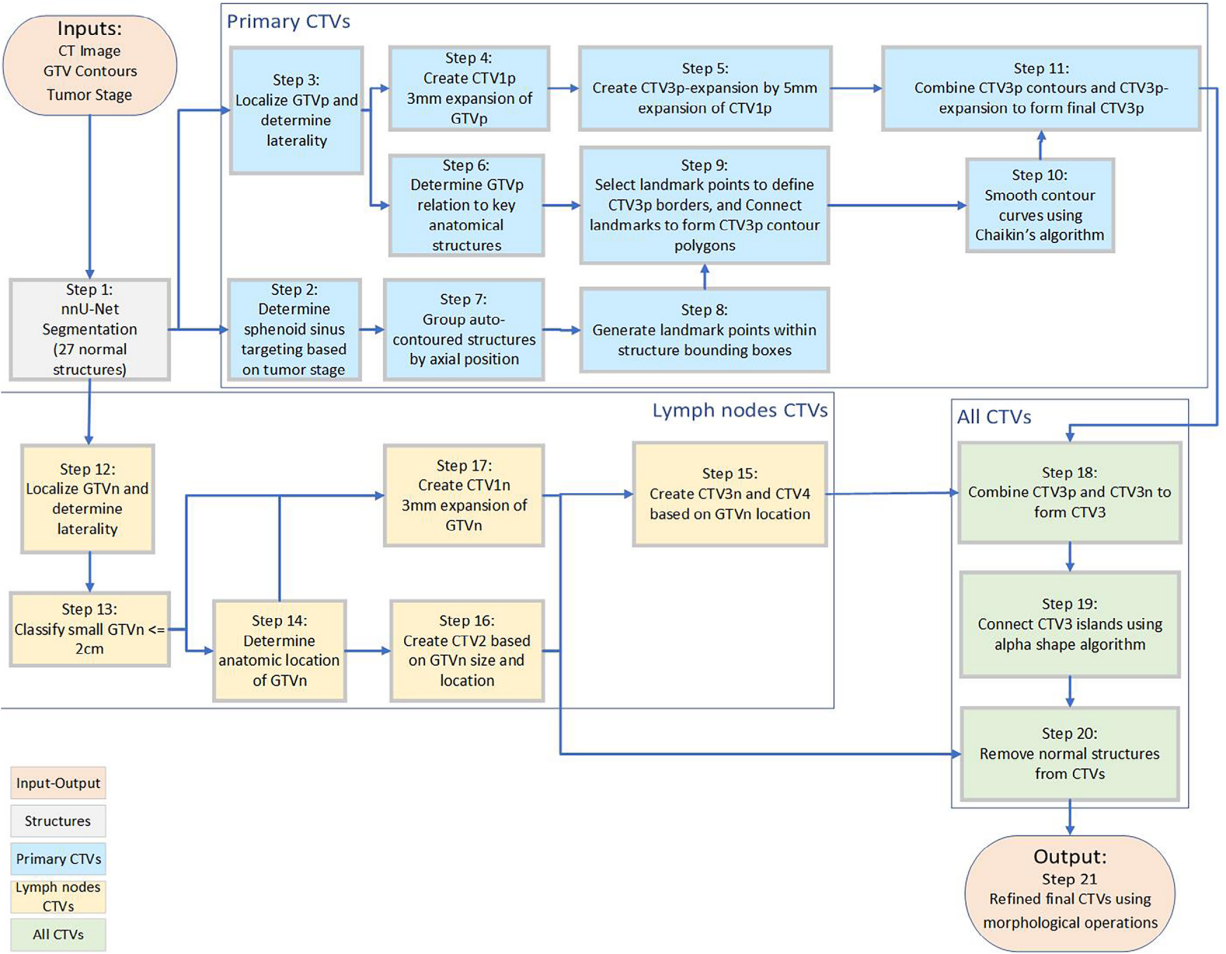
Flowchart of the automated contouring pipeline for nasopharyngeal cancer. The diagram illustrates the process from input data (CT images, GTVp, GTVn, and tumor stage) through various stages of CTV creation, including structure auto‐contouring, preprocessing, landmark identification, and contour refinement, to the final output of CTVs (CTV1, CTV2, CTV3, and CTV4 where applicable).

The set of steps described in this subsection outlines our approach for the automated delineation of CTVs in the treatment of NPC. This approach was applied to the Cohort 2. A detailed flowchart illustrating these steps is provided in Figure 1. We used CT images, primary gross tumor volume (GTVp), nodal gross tumor volume (GTVn), and tumor stage as fundamental inputs.

**FIGURE 2:**
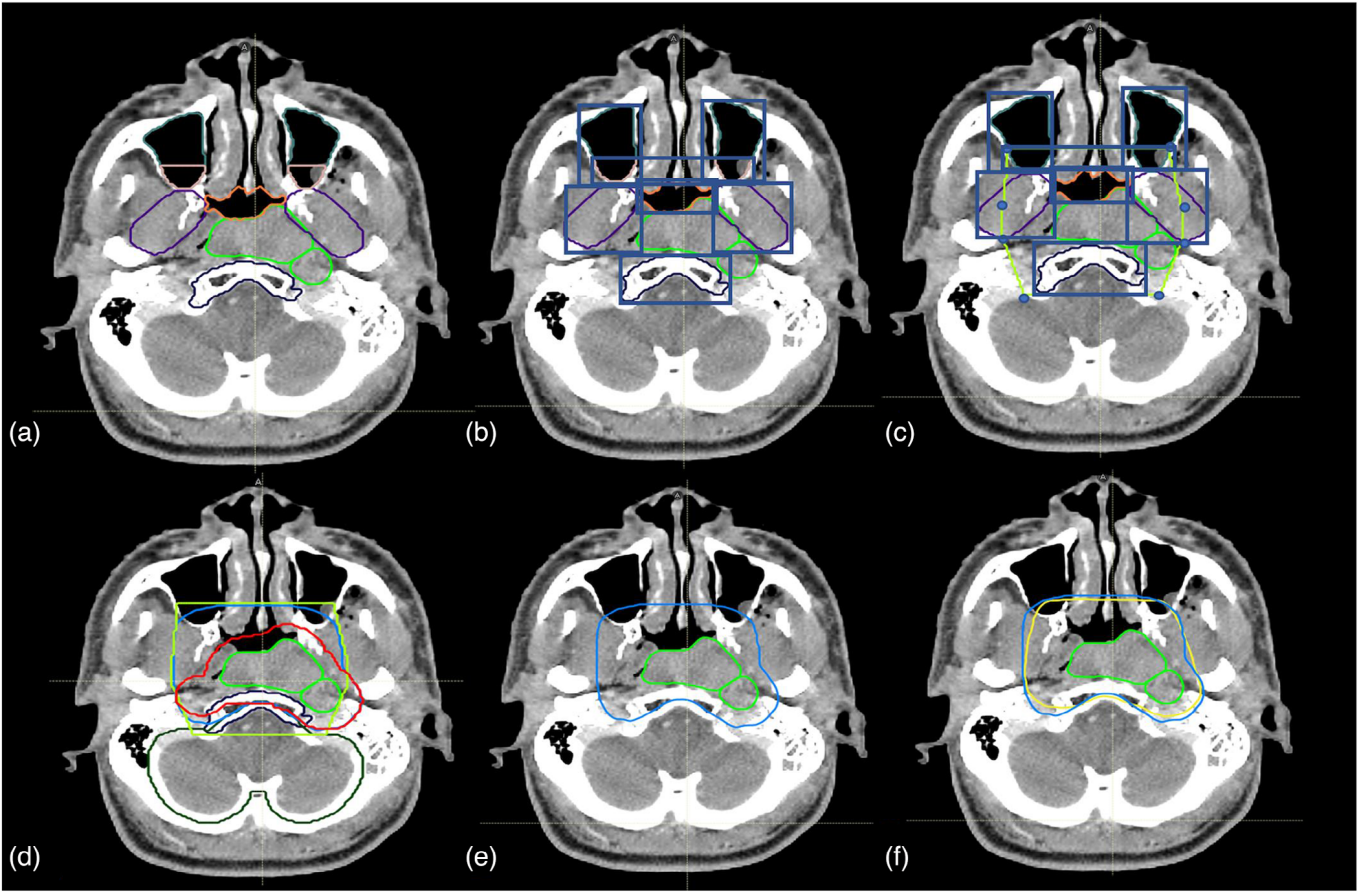
An illustration of the auto‐contouring process. (a) GTV (green) and initial segmentation of anatomical structures. (b) Identification of anatomical structures. (c) Establishment of landmarks, and generation of polygon based on landmarks. (d) Creation of contours with the polygon, and expansion (red) from GTV. (e) Final contour. (f) Comparison between clinical contour (yellow) and automatic contour (blue).

**FIGURE 3:**
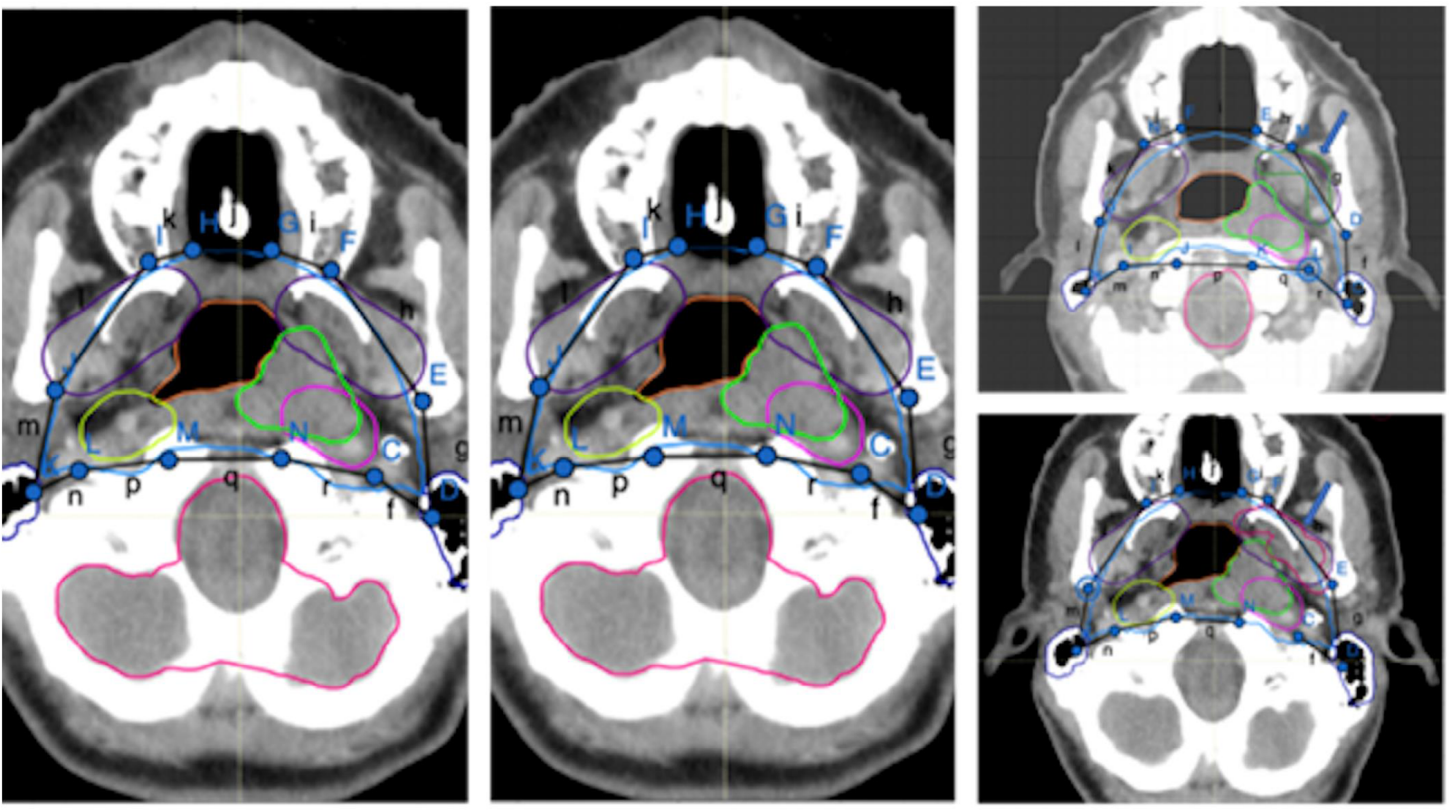
Illustration of the methodology employed for landmark identification. CT images display marked anatomical landmarks (c–e, etc.) connected by colored lines to form polygons, delineating targeted regions for radiation therapy. The lowercase letters correspond to the segments between each landmark point.

**FIGURE 4:**
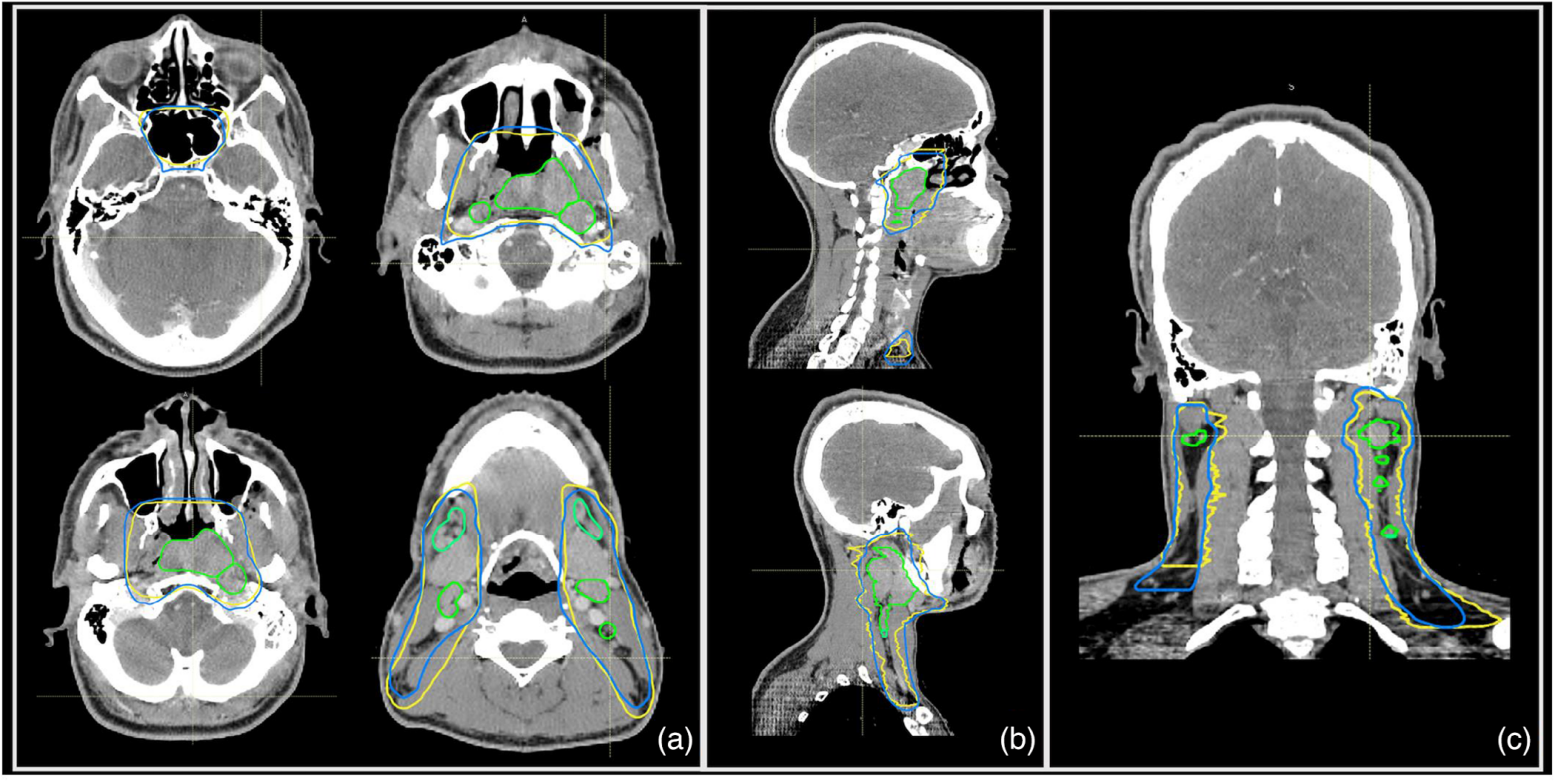
Examples of the clinical contours (yellow) versus auto‐delineated contours (blue). (a) Axial view. (b) Sagittal view. (c) Coronal view.

1. Auto‐contour structures: This procedure systematically auto‐contours 27 vital structures for constructing the CTV. The contouring structures, located in the H&N region, such as the central nervous system, and supporting structures including the brain, clivus, skull base, C1 and C2 vertebral bodies, and spinal cord. Sensory organs and associated structures encompass the left and right orbits, optic nerves, and optic chiasm. Respiratory and oropharyngeal structures consist of the nasopharynx, maxillary sinuses, and sphenoid sinus. Bone and cartilage structures incorporate the hyoid bone and cricoid cartilage. The lymphatic system covers lymph nodes, and the facial and neck muscles and glands comprise pterygoid fossae, parotid glands, and larynx.
2. Sphenoid sinus targeting: Entire sphenoid sinus or just its inferior section may be targeted, for stage >2 or ≤2, respectively. The process begins by calculating the sphenoid sinus's volume to identify its geometric center, which acts as the centroid in the 3D space. The centroid's longitudinal coordinate is located, and a plane is created at this level in the axial axis, dividing the sphenoid sinus into two halves. This division allows for the identification of the inferior section of the sphenoid sinus.
3. Localization of the GTVp using the sagittal plane: A sagittal plane is used to bisect the 3D image into two symmetrical halves. It is then determined whether the GTVp is confined to one side or extended to both sides.
4. Create CTV1p: The clinical target volume 1 primary (CTV1p) is created by expanding GTVp volumetrically by 3 mm following the protocol (see Table 1). CTV1p represents the high‐dose CTV.
5. Create CTV3p‐expansion: A 5‐mm volumetric expansion from CTV1p, named CTV3p‐expansion, is created following the protocol guidelines (see Table 1).
6. GTVp localization: In the GTVp localization phase, we identified the GTVp's location within the nasopharynx, examining its relation to structures such as the clivus, skull base, pterygoid fossae, sphenoid sinus, nasal cavity, and maxillary sinuses. The findings from this step, along with items 7 and 8, directed our landmark selection in item 9 for defining treatment areas.
7. Axial positioning and grouping of structures using longitudinal coordinates: The longitudinal coordinates of each structure is used to group the structures as detailed in Table 2, and visual representation can be seen in Figure 2(a).
8. Generation of evenly spaced points within bounding boxes for contoured structures: For each contoured structure, rectangular bounding boxes are established to demarcate their spatial boundaries. Within these boxes, points are systematically spaced along the x and y axes, using a grid pattern of 7 by 7, to ensure uniform distribution. This method facilitates a comprehensive and detailed mapping of each region of interest (Figure 2b,c).
9. Landmark identification for CTV3p: To determine the intermediate‐dose CTV (CTV3p), key points within the treatment area are identified and selected to establish essential landmarks. For example, for the maxillary sinuses, landmarks are selected at points within the 1/4 anterior section, specifically along the borders of the box that are determined in item 9 in axial slices. In the clivus, points at the corners of the box in its most anterior part are chosen. For the RP lymph nodes and pterygoid fossae, landmarks are established anteriorly along the mid‐vertical line of the defined box. The positioning of landmarks in the pterygoid area varies, adapting to the location of the GTV. These points are selected in accordance with the structures present in each axial slice, as detailed in Table 2 (Figures 2c and 3).
10. Curve generation and smoothing: For each axial plane, landmarks are connected with lines to form polygons (Figure 2d). Subsequently, these polygons are refined using Chaykin's algorithm for curve smoothing and polygonal approximation,^29^ enhancing the contour target volume (CTV3p) (Figure 2c,d).
11. Formation of CTV3p by integrating refined contours and expansion elements: The CTV3p is formed by integrating the refined contours from step 10 with the CTV3p‐expansion outlined in item 5. This process merges both elements to create a unified 3D volume designated as CTV3p, ensuring a comprehensive coverage that encapsulates the expanded and refined target areas (Figure 2d).
12. Localization of GTVn: The localization of GTVn is followed by a procedure similar to that outlined in item 3 for the GTVp. A sagittal plane is employed to bisect the 3D image into two symmetrical halves, facilitating the determination of GTVn distribution – whether it is confined to one side or extended to both. Boolean variables are assigned to each half to denote the presence or absence of GTVn, ensuring a systematic and comprehensive assessment of their spatial distribution.
13. In the comparative analysis of GTVn volumes against sphere diameter criteria, we classify treatment regions, specifically for small‐volume lymph nodes with a GTVn ≤2 cm. This is crucial in areas like level IB, where it guides the decision between CTV1n or CTV2 classifications in step 16.
14. Anatomical orientation and categorization of GTVn and lymph node structures: For each volume within the GTVn, the criteria established in step 13 are applied to determine their specific anatomical orientation, categorizing them as either left‐ or right‐sided. This method is applicable to lymph node structures at levels Ib, II, III, IV, Va, and Vb, identifying their position on either side. The guidelines encompass at‐risk nodal levels, including the upper deep jugular, subdigastric, midjugular, low jugular, supraclavicular regions, upper‐ and mid‐level V, RP areas, and bilateral level IB when directly involved.
15. Creation of CTV3n and CTV4 using results from steps 13 and 14: CTV3n and CTV4 are created by integrating results of steps 13 and 14, aligning with the scenarios outlined in Table 3 (Scenarios 1−9). This process involves spatial analysis and volume measurements of GTVn to accurately define these CTVs. Notably, CTV4 may be used for single intensity‐modulated radiation therapy plans on heminecks that do not have grossly involved low‐lying neck nodes in Level IV, VB, and supraclavicular nodes.
16. Formation of CTV2 from steps 13 and 14: Following the results from steps 13 and 14, CTV2 is created to align with the different scenarios outlined in Table 4 (Scenarios 1−3). This step involves synthesizing the spatial and volumetric data of GTVn for accurate CTV2 formation.
17. Creation of CTV1n: The CTV1 for nodes (CTV1n) is formulated in accordance with the contouring guidelines, integrating insights from our earlier localization and volume determination processes (items 13 and 14). Following these guidelines, CTV1n is created by adding a margin of 3 mm to each GTVn. However, in line with the flexibility allowed by the guidelines, this margin is automatically adjusted as needed, potentially reduced to as low as 0 mm. Such adjustments are made in cases where tumors are adjacent to critical structures. The process involves a thorough analysis of GTVn positioning (step 13), and a careful evaluation of their volumes (item 14).
18. Combine CTV3p and CTV3n to form CTV3: The CTVs for primary tumors (CTV3p) and nodes (CTV3n) are merged, resulting in the formation of a unified CTV, designated as CTV3 (Figure 4).
19. Join islands within CTV3 using Alphashape algorithm: During the formation of CTV3, a number of discrete regions, akin to islands, emerge. Some of these regions are isolated from the main body of CTV3. The Alphashape algorithm is employed to seamlessly integrate these regions into the larger CTV3 structure. This approach ensures the inclusion of smaller nearby areas, effectively creating a unified, non‐convex shape. The use of the Alphashape algorithm allows for the comprehensive encapsulation of these diverse and irregularly shaped regions, resulting in a connected and complete volume representation of CTV3.^30^, ^31^, ^32^
20. Remove normal structures from targets: The brain, spinal cord, optic nerves, chiasm, orbits, vertebral columns, vertebral bodies, hyoid, cricoid, and mandible are subtracted from the CTVs (CTV1, CTV2, CTV3, and CTV4).
21. Refinement of final contours through morphological operations: Morphological operations are applied as a post‐processing step to refine the final contours. These operations included binary opening, binary closing, removal of small holes, and elimination of minor contours. This refinement enhanced the clarity of the contours. Examples of this process and the resultant contours are illustrated in Figures 2 and 4

**TABLE 1:** The protocol guidelines.

Description	Treatment specification / Margin adjustment
**High dose clinical target volume** **(CTVp1)**	
Margin from GTVp	GTVp + 3 mm
Minimal margin if tumor is in close proximity to critical OARs	GTVp + 0 mm
**High dose clinical target volume** **(CTVn1)**	
Margin from GTVn	GTVn + 3 mm
**Intermediate dose clinical target volume (CTVp2)**	
Margin from GTV	GTVp + 8 mm + whole NP
Nasal cavity – Posterior part	1/4
Maxillary sinuses – Posterior part	1/4
Posterior ethmoid sinus	Not stated
Skull base	Cover foramina oval and rotundum + rotundum lacerum and petrous tip
Cavernous sinus	If T3−4 (involved side only)
Pterygoid fossae	+ Included for all cases
Parapharyngeal spaces	+ Included for all cases
Sphenoid sinus	Only the inferior section is targeted for T1–T2 tumors, and the whole tumor is targeted for T3–T4 tumors
Clivus	1/3 of the clivus is targeted if there is no tumor invasion, and the whole structure is targeted if there is invasion
Minimal margin if tumor in close proximity to critical OARs	GTVp + 1 mm
**Intermediate dose clinical target volume (CTVn2)**	
Margin from GTVn	GTVn + 5 mm
Lymph nodes – bilateral	+ Included for all cases
RP, level II, III, & Va	+ Included for all cases
Level Ib	Optional if T1/2N0
**Low dose clinical target volume**	+ Included for all cases

Abbreviations: GTVp, primary gross tumor volume; GTVn, nodal gross tumor volume; OARs, organs at risk; NP, nasopharynx; RP, retropharyngeal.

**TABLE 2:** Grouping of delineated structures based on axial positioning using longitudinal coordinates.

Group number	Structures included
Group 1	Left and right retropharyngeal lymph nodes as well as left and right pterygoid fossae
Group 2	Left and right mastoid, left and right retropharyngeal lymph nodes, left and right pterygoid fossae, and nasopharynx
Group 3	Left and right retropharyngeal lymph nodes, left and right pterygoid fossae, and nasopharynx
Group 4	Clivus, left and right pterygoid fossae, and nasopharynx
Group 5	Clivus, left and right pterygoid fossae, nasopharynx, and maxillary sinus
Group 6	Clivus, maxillary sinus, and sphenoid sinus
Group 7	Maxillary sinus and sphenoid sinus
Group 8	Sphenoid sinus

*Note*: This table presents the organization of structures into groups based on their locations along the axial axis, using longitudinal coordinate analysis.

**TABLE 3:** Clinical target volume (CTV3n) construction for different scenarios based on the protocol guidelines.

Scenario	Conditions	CTV3n and CTV4 construction
1	No GTVn present	Combine lymph nodes II, III, and Va to form CTV3n. Add lymph nodes IV and Vb to create CTV4.
2	GTVn present on both sides of the neck but not localized in lymph nodes IV or Vb	Merge lymph nodes Ib, II, III, and Va to create CTV3n. Add lymph nodes IV and Vb to create CTV4.
3	GTVn present on the left side of the neck but not localized in lymph nodes IV or Vb	Combine lymph nodes Ib L, II, III, and Va to create CTV3n. Add lymph nodes IV and Vb to create CTV4.
4	GTVn present on the right side of the neck but not localized in lymph nodes IV or Vb	Combine lymph nodes Ib R, II, III, and Va to create CTV3n. Add lymph nodes IV and Vb to create CTV4.
5	GTVn present on both sides of the neck and localized in both sides of lymph nodes IV or Vb	Combine lymph nodes Ib, II, III, IV, Va, and Vb to create CTV3n.
6	GTVn present on both sides of the neck but localized only on the left side of lymph nodes IV or Vb	Combine lymph nodes Ib, II, III, Va, IV L, Va, and Vb L to create CTV3n. Add lymph nodes IV R and Vb R to create CTV4.
7	GTVn present on both sides of the neck but localized only on the right side of lymph nodes IV or Vb	Combine lymph nodes Ib, II, III, Va, IV R, Va, and Vb R to create CTV3n. Add lymph nodes IV L and Vb L to create CTV4.
8	GTVn present only on the left side of the neck and also localized on the left side of lymph nodes IV or Vb	Combine lymph nodes Ib L, II, III, Va, IV L, Va, and Vb L to create CTV3n. Add lymph nodes IV R and Vb R to create CTV4.
9	GTVn present only on the right side of the neck and also localized on the right side of lymph nodes IV or Vb	Combine lymph nodes Ib R, II, III, Va, IV R, Va, and Vb R to create CTV3n. Add lymph nodes IV L and Vb L to create CTV4.

**TABLE 4:** Clinical target volume (CTV2) construction based on different conditions.

Scenario	Conditions	CTV2 construction
1	Using the procedure described in item 14, we determined the size of GTVn in the Ib lymph node, identifying if its diameter was less than 2 cm.	Expand the GTVn by 3 mm to construct CTV2.
2	GTVn is positioned in lymph node IV or Vb	Expand the GTVn by 3 mm and add to CTV2.
3	Both scenarios 1 and 2 apply	Amalgamate the outcomes from both scenarios to generate CTV2.

### 2.5 Evaluation of the auto‐contouring tool

The performance evaluation of the auto‐contouring tool based on the protocol guidelines was conducted using quantitative and qualitative analyses.

Quantitative evaluation was conducted by comparing auto‐generated and manual contours, using the dice similarity coefficient (DSC) and the mean surface distance (MSD).^33^, ^34^, ^35^

Two radiation oncologists, each with specialized expertise in H&N cancers and extensive experience in treating NPC, conducted independent reviews of the clinical acceptability of the auto‐contours. Utilizing a previously established 5‐point Likert scale,^20^ they separately evaluated and scored the contours, offering insights into their clinical usability. Qualitative assessments used a 1‐to‐5 Likert scale, where 1 marked unusable contours and 5 indicated contours can be “use‐as‐is”. Detailed scoring criteria are provided in Table 5.

**TABLE 5:** Likert scale scoring criteria for clinical acceptability assessment of auto‐contoured clinical target volumes (CTVs) by independent physicians.

Likert scale	Explanation for this study
5	Strongly agree: Use as is (i.e., clinically acceptable and could be used for treatment without change)
4	Agree: Minor edits that are not necessary. Stylistic differences but changes that are not clinically important. The current contours/plan are acceptable.
3	Neither agree nor disagree: Minor edits that are necessary. Minor edits are those that the review judges can make in less time than starting from scratch or are expected to have minimal effect on treatment outcome.
2	Disagree: Major edits needed. This category indicates that the necessary edits are required to ensure appropriate treatment, and sufficiently considerable that the user would prefer to start from scratch.
1	Strongly disagree: Unusable. This category indicates that the quality of the automatically generated contours or plan are so poor that they are unusable.

## 3 RESULTS

### 3.1 Segmentation performance of the structures necessary to create the CTVs

In our evaluation of the automated anatomical structures, we compared the automated and reference contours to assess the performance of our automated contouring process, as shown in Table 6 provides a comprehensive breakdown of these metrics across various structures.

**TABLE 6:** Comparative analysis of DSC and MSD across various structures.

Structures	Mean DSC	Mean (mm)	MSD
Brain	0.99 ± 0.00	0.5 ± 0.1	
Clivus	0.89 ± 0.04	0.9 ± 0.3	
Cricoid	0.82 ± 0.14	1.2 ± 1.1	
Hyoid	0.86 ± 0.10	0.7 ± 0.5	
Left mastoid	0.83 ± 0.07	1.1 ± 0.4	
Right mastoid	0.81 ± 0.10	1.3 ± 0.5	
Maxillary sinus	0.94 ± 0.03	0.7 ± 0.2	
Nasopharynx	0.89 ± 0.06	1.1 ± 0.4	
Left orbit	0.94 ± 0.02	0.9 ± 0.2	
Right orbit	0.94 ± 0.02	0.9 ± 0.2	
Pterygoid fossae	0.88 ± 0.02	1.3 ± 0.2	
Sphenoid sinus	0.91 ± 0.09	0.8 ± 0.6	

### 3.2 Segmentation performance protocol‐based CTVs

**FIGURE 5:**
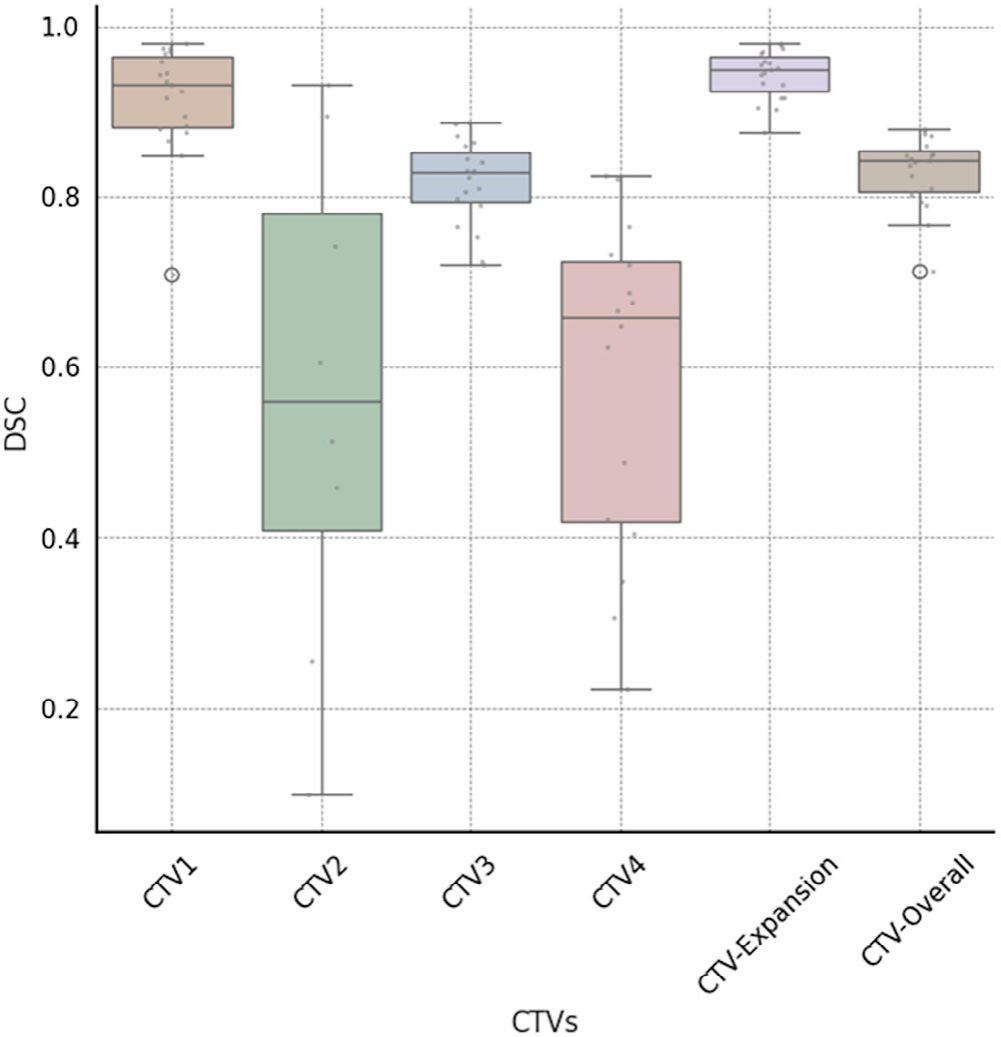
Box and whisker plots of dice similarity coefficient (DSC) distance between ground‐truth and automatically generated contours by our tool CT images. For this analysis, CTV3 represents the aggregate of CTV3p and CTV3n, while CTV1 is constituted by the sum of CTV1p and CTV1n. The central line represents the median value. The border of the box represents the 25th and 75th percentiles. The outliers are represented by circles markers.

**FIGURE 6:**
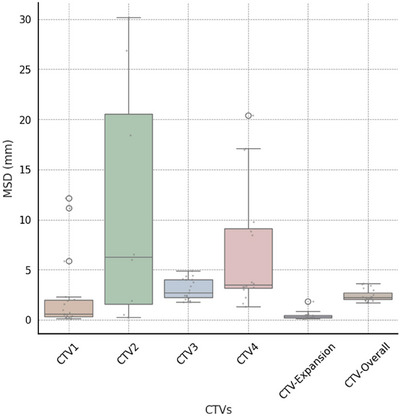
Box and whisker plots of mean surface distance (MSD) distance between ground‐truth and automatically generated contours by our tool's CT images. For this analysis, CTV3 represents the aggregate of CTV3p and CTV3n, while CTV1 is constituted by the sum of CTV1p and CTV1n. The central line represents the median value. The border of the box represents the 25th and 75th percentiles. The outliers are represented by circles.

**FIGURE 7:**
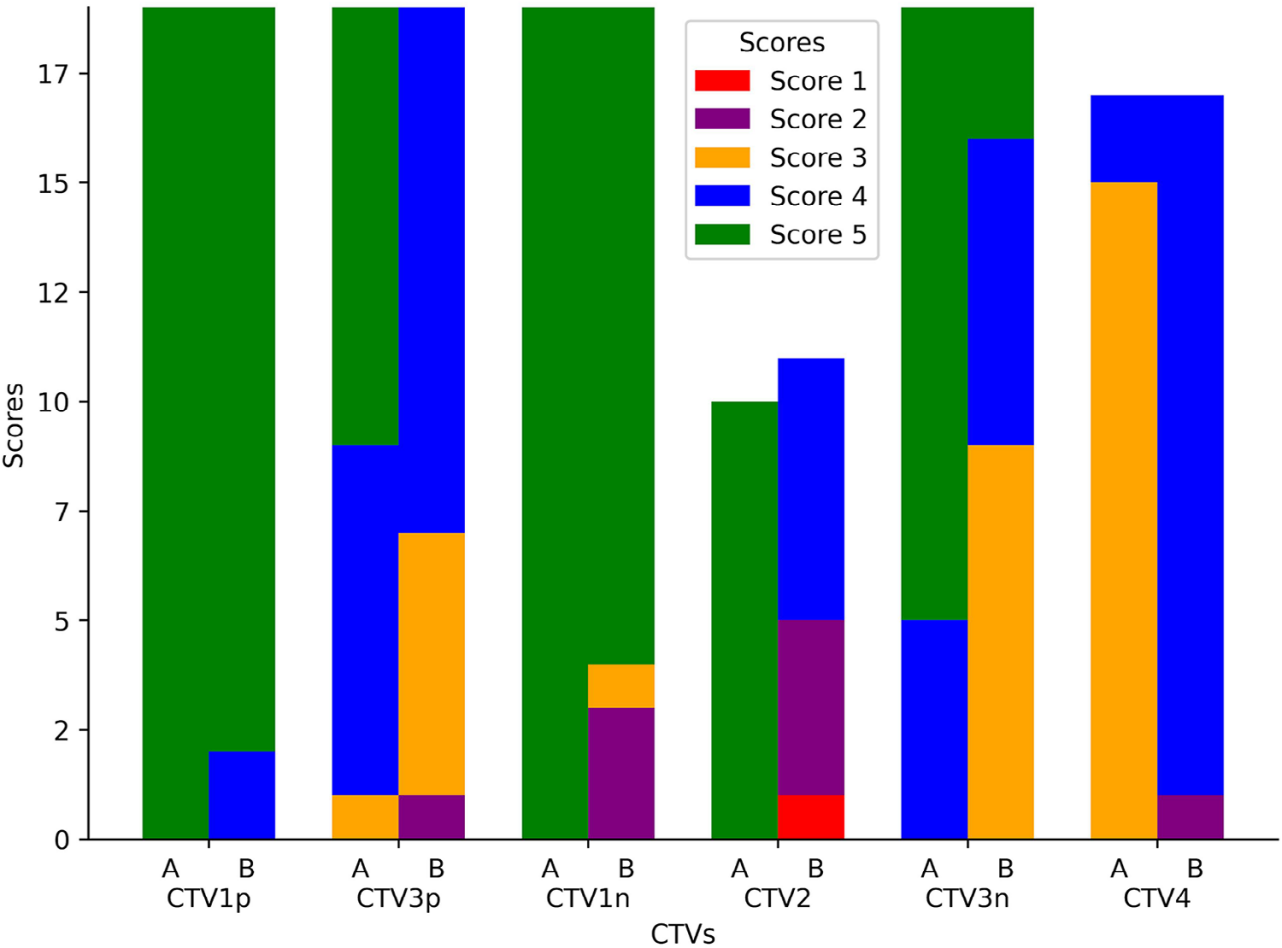
Stacked bar plots of contour scores by Physician A and Physician B. Unlike previous figures that presented aggregate data for CTV3 and CTV1, this figure provides a detailed breakdown. Here, CTV3 is dissected into CTV3p and CTV3n, and CTV1 is segmented into CTV1p and CTV1n, offering a more granular view of the contour scores for these subdivisions.

In the evaluation of Cohort 2 (contoured per protocol guidelines) using an auto‐contouring tool, the tool achieved with the CTV‐Expansion (i.e., the combination of CTV1 and CTV2) a DSC of 0.94. This deviation from the ideal score of 1.0 is because this is not just a simple expansion and is attributed to the manual adjustments made to the clinical contours and the automatic modifications applied by our tool. These modifications are typically necessary when tumors are adjacent to critical structures. Moreover, the MSD of 0.4 mm, again not achieving the ideal zero, for the same reasons affecting the DSC in the CTV‐Expansion. For CTV‐Overall (the outer envelope of all the CTV contours), the tool achieved a DSC of 0.83 and an MSD of 2.4 mm, (Figures 5 and 6). Variations were observed across different CTVs. In the review by two independent H&N physicians, 90% of the auto‐contours were rated as clinically suitable, often requiring little to no modification (Figure 7).

Using the DSC, CTV‐Expansion had the highest average DSC at 0.94. In contrast, CTV2 had the lowest DSC average at 0.56, with noticeable variability in DSC values, particularly for CTV2 and CTV4 (Figures 5 and 6).

CTV‐Overall had an average MSD of 0.4 mm, demonstrating a high level of precision, whereas CTV2 showed a higher average MSD of 11.3 mm, indicating greater variability in its precision metrics. This highlights the differences in segmentation accuracy across various CTVs (see Figures 5 and 6).

In a separate consideration, interpretation differences, particularly with CTV2 and CTV4, contribute to the observed variability. The decision to merge CTVs, as in the case of combining CTV1 and CTV2 into CTV‐Expansion, appears to reduce such inconsistencies. This finding suggests that strategic combinations of CTVs can enhance overall accuracy in segmentation.

The observed variations can be attributed to subjective interpretation differences, particularly at the borders between CTV2 and CTV1n, where the choice between classifications is left to the discretion of the physicians. This leads to differing decisions on whether a specific area should be classified under one category or the other, causing variability in segmentation. A similar situation arises with CTV4 and CTV3n, where distinguishing their boundaries presents comparable challenges. Further clarification on these interpretation differences, particularly in the context of the comparative analysis between the two physicians, will be provided in the discussion section. These differences are hidden when we consider the overall CTV.

To subjectively assess compliance with protocol guidelines, two physicians quantitatively assessed CTVs specifically for the cohort 2 (which was contoured per protocol guidelines). Figure 7 provides a visual representation of the evaluations made by Physicians A and B, using a stacked bar plot to depict the differences in contour scores. Physician A consistently rated all CTVs with a score of at least 3, suggesting minor necessary edits.

Physician B's evaluations displayed more variation; 90% of their ratings for all CTVs were 3 or higher, but only 54% of ratings for specific CTVs, such as those for CTV2, met this mark. Physician B reviewed 11 cases classified as CTV2, while Physician A reviewed only 10 such cases. This discrepancy occurred because Physician B reclassified one case from CTV1n to CTV2. Consequently, Physician B's review included this additional case, resulting in a total of 11 CTV2 cases, compared to the 10 reviewed by Physician A.

In the lower neck region, Physician A proposed minor modifications for cases where CTV4 was deemed overly large, favoring limited muscular coverage. Conversely, Physician B did not think of a need for such minor edits, indicating different approaches to regional coverage.

## 4 DISCUSSION

The method proposed in this study combines deep learning with a clinical rule‐based algorithm to create an automated strategy for CTV contouring that more closely mimics the natural physician contouring process. It is more directly based on clinical guidelines rather than on variable human‐drawn CTV contours, so it should be relatively straightforward to adapt to new protocols. Beyond being flexible, the method produces CTV contours in close agreement with contours drawn by experts, as demonstrated by our findings of average DSCs of 0.94 for CTV‐Expansion and 0.83 for CTV‐Overall and MSDs of 0.4 mm for CTV‐Expansion and 2.4 mm for CTV‐Overall. These outcomes were further validated by the assessments of two H&N physicians, who scored 90% of the contours as a three or greater, indicating they are clinically satisfactory and generally require only minor edits.

Although impact of image quality on auto‐contouring performance was not specifically addressed in this work, Huang et al.^36^ conducted a study on this matter. They showed that auto‐contouring performance was minimally affected by slice thicknesses up to 3 mm, pixel sizes between 0.49 and 1.17 mm, and varying CT dose levels, which are within the range of the clinical protocols of the current study. They found that reducing the CT dose had a minimal effect on auto‐contouring accuracy, with the CNN‐based method being less sensitive to dose level changes compared to the multiatlas‐based method. These findings suggest that the autocontouring tool described in the current work can perform robustly within the range of typical clinical imaging parameters.

Furthermore, the auto‐contouring tool in this work addresses concerns related to tumor heterogeneity and anatomical variations by using GTVs and tumor stage as inputs, allowing the planner to account for the heterogeneity of the GTV before initiating the auto‐contouring process. The use of MRI, particularly during the diagnostic workup and simulation, can help guide GTV delineation by identifying features such as pterygoid involvement, clivus involvement, or perineural spread at the skull base. These factors can influence the extent of CTV coverage, as mentioned in the NRG‐HN001 protocol. By relying on input GTVs and adhering to the protocol guidelines, the tool in this work effectively addresses concerns related to tumor heterogeneity and anatomical variations.

Our landmark approach, emphasizing rule‐based methodologies, offers notable flexibility for adapting to different guidelines. In contrast to the methods employed in studies that focus on dataset‐centric, model training for CTVs^13^, ^14^, ^15^ ^,^ our study adopts a different approach. This method resulted in a DSC of 0.83 and an MSD of 2.4 mm, showcasing the feasibility of reliable outcomes without reliance on extensive training datasets for the CTVs. These results, together with positive clinician feedback, highlight the potential efficacy of guideline‐adherent, landmark‐based auto‐contouring in NPC treatment.

The protocol defines fixed margins for CTV expansions, such as 3 mm for CTV1p and CTV1n. However, the protocol also allows for margin reduction down to 0 mm when tumors are adjacent to critical structures. We recognize that using these fixed margins may not always consider individual patient characteristics or specific clinical scenarios. To address this, our algorithm allows for flexible margin adjustments, as described in step 17 of the auto‐contouring process, enabling the margins to be reduced to as low as 0 mm when necessary, in line with the protocol guidelines. Future research should explore the impact of personalized margin expansions on treatment volume estimation.

Although we did not perform interobserver variability studies and instead focused on geometric comparisons, other studies have shown that autocontouring can help mitigate interobserver variability and improve efficiency in the treatment planning process, even when some manual editing is required. Maduro Bustos et al.^37^ found that an AI‐based autocontouring algorithm for OAR in the pelvis, thorax, and H&N regions yielded substantial time‐saving efficiency, ranging from 67% to 84%, despite the need for some manual refinement. Similarly, McCarroll et al.^38^ reported that 87% of autocontours for H&N normal structures were clinically acceptable without edits, and when minor edits were needed, the autocontours still served as reliable starting points.

Regarding the transition between CTV3p and CTV3n, Physician A generally scored cases as clinically acceptable or needing minor changes, but Physician B scored cases with more variation. Namely, the scores from Physician B showed a near‐even split between cases requiring minor edits and those that did not. This variation highlights the individualized aspect of clinical judgment. The distinct difference in their evaluations of CTV3p, with Physician A at 95% approval versus Physician B at 63%, notably reflects differing interpretation of the contouring guide.

Liu et al.^39^ demonstrated that while target volumes delineated by different physicians had high similarity, significant differences were found in the maximal distances between outer contours, particularly in patients with advanced T stages. These deviations led to significant differences in dose distributions. The autocontouring tool described in the current study, which incorporates a rule‐based algorithm following the NRG‐HN001 protocol, aims to mitigate interobserver variability by providing consistent contours based on established guidelines. The high acceptance rates of our autocontours by multiple physicians suggest that the tool can effectively generate clinically acceptable contours, even in the presence of interobserver variability. Our autocontouring tool serves as a good starting point for manual refinement treatment planners, reducing effort required for contouring.

The observed variance in clinical acceptability of our auto‐contours, particularly the 10% discrepancy where one physician required major edits while the other found the contours usable, aligns with the challenges in defining “clinical acceptability”’ highlighted by Baroudi et al.^40^ This variability reflects the subjective nature of contour assessment in radiation therapy, which exists in both manual and automated processes. Despite this discrepancy, our tool's overall high acceptance rate (90% deemed clinically suitable) suggests its value as a consistent starting point for treatment planning. Baroudi et al. emphasize that automated tools can help standardize contouring practices and reduce interobserver variability over time.^40^ In this context, our auto‐contouring tool provides a standardized foundation that could improve workflow efficiency, even when some edits are necessary. The observed differences in physician assessments underscore the need for continued refinement of auto‐contouring algorithms, potentially incorporating more detailed protocol‐specific rules to further enhance clinical acceptability and reduce major editing requirements.

It is important to note that the cases where Physician B scored below acceptability were primarily related to disagreements between CTV1n and CTV2 classifications, rather than issues with the overall contour quality. This discrepancy stems from the interpretability of the protocol rules, particularly in border regions where the decision between CTV2 and CTV1n is less clear‐cut. Notably, 80% cases that were deemed not acceptable occurred in patients with N3 stage disease, highlighting the increased complexity and variability in interpretation for advanced nodal disease. Interestingly, the auto‐contouring tool performed very well for primary tumor stages T2, T3, and T4, with the main challenges arising in the delineation of lymph node regions. This observation underscores the difficulties in standardizing contours for extensive lymph node involvement, where the boundaries between different CTV levels become more intricate and subject to clinical judgment, despite accurate handling of the primary tumor extent.

Interestingly, even in cases where physicians identified a need for modifications, the quantitative metrics (DSC and MSD) remained relatively high. For instance, in the cases requiring minor edits, we observed DSC values ranging from 0.79 to 0.97 and MSD values between 0.2 and 3.5 mm. This observation suggests that while these metrics are valuable for overall assessment, they may not fully capture the nuances of clinical usability. High DSC and MSD scores do not necessarily guarantee that a contour will be clinically acceptable without modification. This discrepancy highlights the importance of combining quantitative metrics with expert clinical evaluation to comprehensively assess auto‐contouring performance.

A discrepancy arose in a case involving GTVn in the upper border of a patient's lower left neck. Physician A considered the GTVns appropriately addressed with different treatments based on size distinctions, suggesting CTV3 over CTV4 for a specific zone. In contrast, Physician B identified inconsistencies, advocating for a uniform approach with CTV4.

## 5 CONCLUSIONS

This study assesses an innovative auto‐contouring tool for NPC treatment, focusing on anatomical structures. The tool showed good agreement with clinical contours in both quantitative and subjective evaluations, suggesting its potential to effectively aid in radiation therapy planning and protocol standardization.

## AUTHOR CONTRIBUTIONS

**Carlos Sjogreen**: Conceptualization; methodology; software; validation; formal analysis; investigation; resources; writing—original draft; writing—review & editing; visualization. **Tucker J. Netherton**: Conceptualization; methodology; formal analysis; resources; writing—review & Editing. **Anna Lee**: Formal analysis; data curation; validation; writing—review & editing. **Moaaz Soliman**: Formal analysis; data curation; writing—review & editing. **Skylar S. Gay**: Formal analysis. writing—review & editing. **Callistus Nguyen**: Software. **Raymond Mumme**: Software. **Ivan Vazquez**: Formal analysis; writing—review & editing. **Dong Joo Rhee**: Formal analysis; software. **Carlos E. Cardenas**: Conceptualization; formal analysis; writing—review & editing. **Mary K. Martel**: Funding acquisition. **Beth M. Beadle**: Formal analysis; data curation; validation; writing—review & editing. **Laurence Edward Court**: Conceptualization; investigation; methodology; validation; formal analysis; resources; writing—original draft; writing—review & editing; project administration; funding acquisition.

## CONFLICT OF INTEREST STATEMENT

The authors declare no conflicts of interest.

## Supporting information

Supporting Information
