# Supplementary material for: Landmark‐based auto‐contouring of clinical target volumes for radiotherapy of nasopharyngeal cancer
Source: J Appl Clin Med Phys. 2024 Jul 29;25(9):e14474. doi: 10.1002/acm2.14474 (PMC11492310; doi:10.1002/acm2.14474)
Supplement: Supplementary file 1 — Supporting Information [file ACM2-25-e14474-s001.docx]

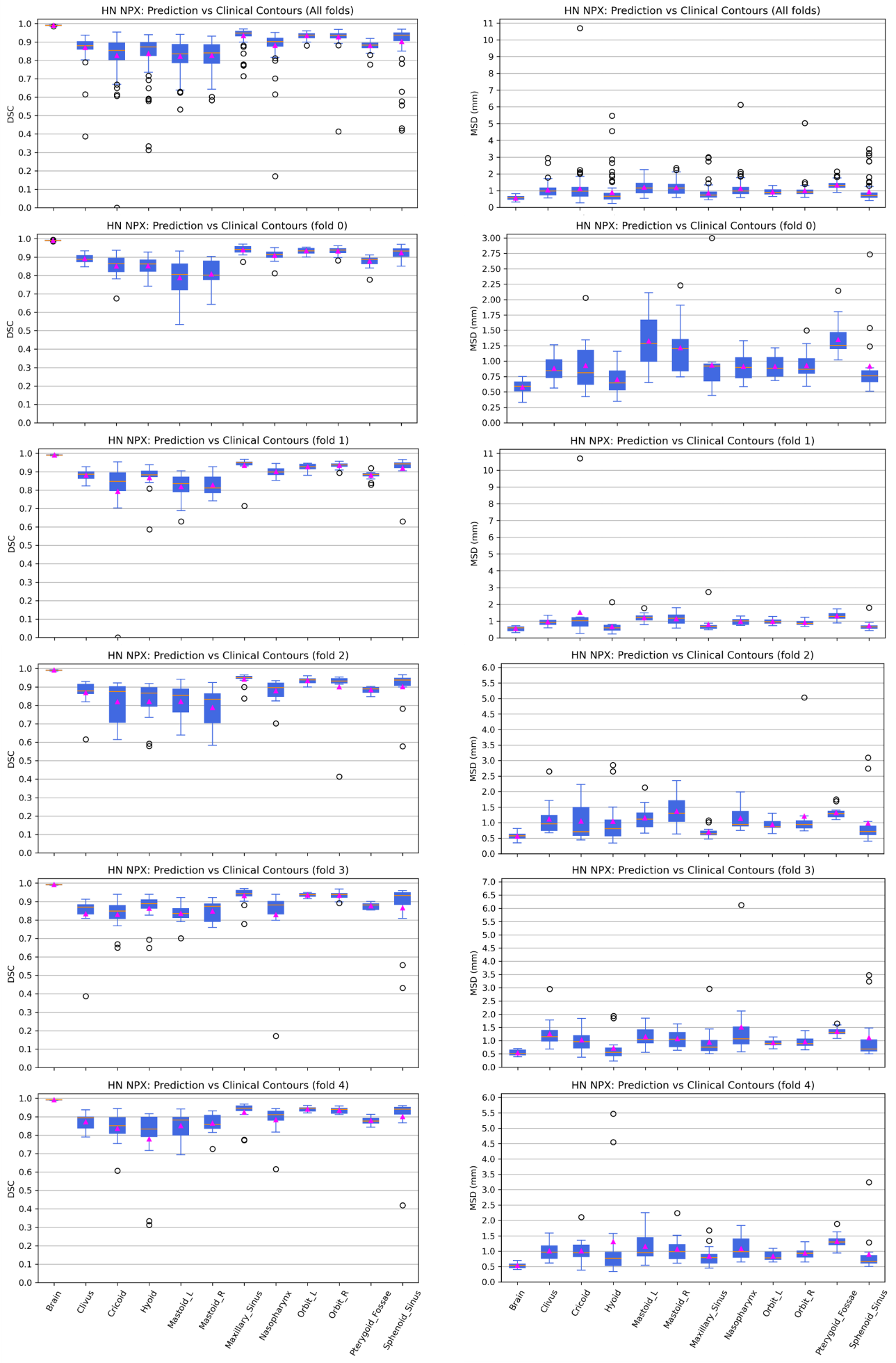


Supplementary Figure 1. Detailed cross-validation results for the deep learning model of nasopharyngeal cancer structures. The figure shows box plots of Dice Similarity Coefficient (DSC, left column) and Mean Surface Distance (MSD, right column) for various anatomical structures. Results are presented for all folds combined (top row) and for each of the five individual folds (subsequent rows). Each box plot displays the median, interquartile range, and outliers for the respective metric across different structures.
